# Copper and zinc from slurry applications shape grassland soil microbial community structure in a long-term experiment

**DOI:** 10.3389/fmicb.2026.1930634

**Published:** 2026-10-14

**Authors:** Coalain McCreanor, Wanqi Jia, Manus Carey, Caroline Meharg, Andrew Alexander Meharg, Jonathan Holland, Selva Dhandapani, Paul N. Williams

**Affiliations:** 1 School of Biological Sciences, Queen's University Belfast, Belfast, United Kingdom; 2 Co-Centre for Sustainable Food Systems, School of Biological Sciences, The Queen's University of Belfast, Belfast, United Kingdom; 3 Agri-Food and Biosciences Institute, Belfast, United Kingdom

**Keywords:** multi-species swards, One Health, potentially toxic elements, soil health, sustainable agriculture

## Abstract

Soil microbes are crucial in maintaining soil and plant health, underpinning agronomic productivity. This study investigates antimicrobial copper- and zinc-laden cow and pig slurry applications on soil microbial community structures in an approximately 50-year long-term slurry experiment on a permanent grassland located in County Down, Northern Ireland. Illumina and PacBio rRNA sequencing (analysing 16S and ITS genera) were used to investigate soil samples (to 15 cm depth) from the experiment’s treatments. Slurry type and application rate were found to alter the soil microbial community structure. The highest slurry application rate resulted in the greatest log-2-fold changes in most relative abundances. Soil pH (ranging from ~6.5 to ~5.8) and carbon content (ranging from ~7% to ~5%) were also significantly elevated in the highest slurry treatments relative to the non-amended control. Soil pH, copper, and zinc concentrations were found to be significant key drivers of soil microbial community shifts (permutation test, *p* < 0.001). More specifically, copper primarily influenced bacterial (16S; *F* ≈ 25) and zinc has the greatest influence on fungal (ITS; *F* ≈ 13) genera. A key finding was that these two community types responded differently to slurry management: Canonical analysis of principal components showed 16S community structure diverged by both slurry type and application rate, whereas ITS community structure was distinguished by application rate alone. Despite this, there is no sign that it has detrimental impacts on grass yield, and indeed, some promoted microbes (*Nitrospira* spp.) were associated with increased productivity. However, the potentially pathogenic organism (i.e., *Fusarium* spp.) observed in all treatments is of concern, given the contaminating mycotoxins they produce. The findings illustrate soil microbial responses to sustained and settled long-term organic-amended soils that could not be observed without the long-term nature of this experiment. These findings indicate that long-term Cu and Zn-loaded slurry application restructures the soil microbial community in significant ways with implications for antimicrobial resistance selection and a hypothesised link to mycotoxin-related food safety for further investigation, within a One Health framework linking soil, plant, animal, and human health.

## Introduction

The temperate maritime climate of the island-of-Ireland supports high sward yields, ensuring a consistent supply of quality, affordable forage, and silage-based animal feeds (McDonnell et al., 2019). This productive grassland agroecosystem currently sustains an island-wide population of approximately 8.5 million cattle and over 2.5 million pigs (Central Statistics Office, Ireland, 2025; Department of Agriculture, Environment and Rural Affairs, 2025). Concurrently, the livestock sector generates substantial quantities of organic fertilisers, with bovine and porcine slurries being particularly significant within pasture-based systems (Köninger et al., 2021; Silva et al., 2025; Girón-Domínguez et al., 2026). The production of these slurries has increased in tandem with considerable growth in animal numbers over recent decades (Central Statistics Office, Ireland, 2017). Permanent grasslands are increasingly re-transitioning towards management practices underpinned by organic-based fertilisers, such as slurries and manures (Silva et al., 2025). Beyond their cost-effectiveness, these amendments reduce dependence on imported synthetic fertilisers and can help alleviate eutrophication pressures (Lima et al., 2025; Xu et al., 2025). Moreover, renewed emphasis on their application is partly driven by their technical potential for soil organic carbon (SOC) sequestration, a key consideration within agricultural policies aimed at achieving carbon net-zero targets (Fornara et al., 2020; Fornara et al., 2016; Zhang et al., 2006).

Soil microbiota play a fundamental role in SOC stabilisation and its long-term persistence (Christie and Beattie, 1989; Bhattacharyya et al., 2022). Microbial residual carbon (MRC) contributes approximately 40% of stable SOC, underscoring the critical importance of these below-ground processes (Wen et al., 2025). However, the extent to which organic amendments influence microbial community structure, MRC formation, and ultimately SOC stabilisation remains insufficiently understood within temperate grassland systems of north-western Europe (Abbruzzese et al., 2021). Evidence from a 50-year grassland field trial in Northern Ireland (NI) has shown that, under high slurry application regimes, microbial biomass declines. Reductions were attributed to elevated zinc (Zn) and potentially copper (Cu) in the soil (Christie and Beattie, 1989). This stemmed from slurries produced by animals administered with pharmacological doses of the metals, as an antimicrobial, for growth-promoting effects (Christie, 1987; Christie and Beattie, 1989). The One Health concept recognises that human, animal, and environmental health are interdependent, with a change in one propagating through the system (Mackenzie and Jeggo, 2019). Under this framework, a long-term elevation of Zn and Cu creates a selective environment for metal-resistant microbes, establishing a soil reservoir of resistance traits that can co-select for broader antimicrobial resistance (Gillieatt and Coleman, 2024).

Zn toxicity is characterised by broad biochemical interference, whereby Zn can competitively bind and displace Mn, Mg, and Fe from a wide set of structural and catalytic enzymes (Imlay, 2013, 2014, 2015; Alqaffaf et al., 2026). It also forms strong bonds with N-, O-, and S- (Karlsson and Skyllberg, 2007; Hussain et al., 2022). As a moderate membrane disruptor, it also induces DNA damage and in the case of nanoparticle species can generate ROS (Singh et al., 2020; Alqaffaf et al., 2026), although in general Zn is considered redox inert (Andreini et al., 2006). Cu is more chemically aggressive, evidenced by its more widespread use as a fungicidal agent (Giller et al., 1998; Lamichhane et al., 2018). It is highly redox active and has a strong metabolic role in electron transfer reactions (Kim et al., 2008). Thus, ROS, membrane permeabilisation, and DNA adducts are consequential damage evoked by excess Cu (Mathews et al., 2013). Peptide, sulfhydryl groups, and cystine-rich proteins all exhibit a high affinity for Cu, forming the mechanistic backbone of cellular detoxification processes (Chatterjee et al., 2020). In soil, Cu is considered more mobile where acidic, hydrolysing more readily than Zn, due to its higher charge density and polarising power (Martínez and McBride, 1998; Agbenin and Olojo, 2004). Interaction chemistries with DOC are also markedly different, with Cu having high-affinity binding and Zn much less so (Zhao et al., 2007). Here, DOC drives the mobility, speciation, and toxicity of Cu, whereas this influence is far less dominant for Zn (Zhao et al., 2007).

The consequences of Cu and Zn exposure for microbial community structure can be dependent on nutrient exposure duration (Shaw et al., 2020). Short-term experiments regularly state this as limiting in their experimental design (Griffiths and Philippot, 2013; Li et al., 2015; Shaw et al., 2020). Observances from Cu and Zn amendments commonly include reductions in microbial biomass and activity, stemming from the homeostatic disturbance and subsequent microbial structural response (Giller et al., 1998; Guarino et al., 2020; Shaw et al., 2020). A 5-year China-based study on red and fluvo-aquatic arable soils, contaminated with varying copper dosages, showed significantly reduced microbial biomass carbon and a community selection towards copper tolerance in bacteria (Li et al., 2015). Similarly, microbial community structure was found to have changed from Zn- and Cu-derived sewage sludge, 11 years since addition on arable and grassland soil for fungi, archaeal, and bacterial taxa (Macdonald et al., 2011). In this study, time effects are overcome, whereby soils have continuously been supplied with regular nutrient treatments for ~50 years, and therefore likely established an enduring microbial structure.

Bacteria, archaea, and fungi define soil function and services (Delgado-Baquerizo et al., 2020; Van Der Heijden et al., 2008; Xu et al., 2019). Unlike shorter-term studies, the impacts on the microbial community structure from five decades of repeated applications of these Cu- and Zn-laden slurries in grassland soils are less understood. The aim of this study was to determine the long-term effects of Cu- and Zn-laden slurry application on soil microbial community structure. Using Northern Ireland’s premier long-term fertiliser experiment for grasslands, the long-term slurry (LTS) trial based at Hillsborough, County Down, we tested three hypotheses:

*H_1_:* that this prolonged exposure has produced a structurally distinct, metal-tolerant microbial community between nutrient treatments.

*H_2_:* that community structure differs by nutrient source and application rate, reflecting the contrasting Cu and Zn loadings of cow and pig slurries.

*H_3_:* that microbial community responses differ between monoculture grassland and multispecies swards (MSS).

## Materials and methods

### Experimental setup and field sampling

The LTS experiment consists of 48 plots of 29.75 m^2^, initially seeded with a perennial ryegrass sward mix, where 8 randomised nutrient treatments were applied yearly since 1970, with 6 replicates per treatment (Christie, 1987; Fornara et al., 2020). With a randomised block design, treatments are of cow and pig slurry applied at 50, 100, and 200 m^3^/ha/year each, a mineral fertiliser (NPK at a rate of 200, 32, and 160 kg/ha/year, respectively), and a non-fertilised control. Northern Ireland’s Department for Agriculture, Environment and Rural Affairs had previously recommended a slurry application rate of 50 m^3^/ha/year for managed grasslands for silage production (Jordan et al., 2007). The higher application rate treatments were included to look at the impact of scenarios where excess slurry was applied, as NI has an oversupply of slurry (Christie, 1987). Excess slurry application is also *ad hoc* approved by the department where warranted. Nutrients are applied 3 times a year (March, May, and July), in equal amounts, with sward cutting preceding top dressings.

In September 2019, 3 replicate plots for each treatment were randomly selected to be ploughed, and MSS reseeded to investigate associated biomass production and other agronomic foraging plants (Holland et al., 2024). As half of the LTS plots are managed under relatively new soil and rhizosphere-disruptive MSS management, this study places emphasis on both the MSS vs. undisturbed treatments and the overall nutrient treatments. The plots were ploughed to a depth of 10-15 cm using a Power Harrow before MSS reseeding. MSS reseeding mix consisted of Ryegrass, White Clover, Plantain, and Chicory and was administered by hand. The soil on which this experiment was conducted was dystric cambisol soil (Holland et al., 2024). Soil samples were collected in October 2021 to a depth of 15 cm using a hand-held auger, to be used for the chemical and microbial investigation. A depth of 15 cm was selected as it represents the junction of microbial activity, nutrient topdressing exposure, alongside plant activity.

### Analytical methods

Collected soils were frozen (−20 °C), freeze-dried (Christ Alpha1-4LD Plus, Osterode am Harz, Germany) for 24 h at −50 °C in pressure 0.2mBar until dry and 2 mm sieved, with grass and roots removed. Samples were finely pulverised in a ball mill (Retsch PM 100, Haan, Germany) equipped with zirconium oxide-lined chambers and grinding balls for metal extraction. Total carbon percentage was measured on a LECO Truman CN Analyser on air-dried soil (at 102 °C) passed through a 150 μm sieve, combusted in total oxygen conditions (Fornara et al., 2016). Soil pH was determined by a 1:2.5 soil-to-distilled-water ratio mix (Murphy et al., 2005). Total soil carbon and pH were measured from the February 2021 LTS soils before the first sward cut. pH on the LTS is maintained between 5 and 7.2 (Jia et al., 2024), and total carbon is more reflective of year-round values as it contains recalcitrant fractions (Fornara et al. 2020; Fornara et al., 2016). Dry grass yield (kg/ha) of the September 2021 harvest from each of the plots was also obtained by the same method as described in Christie and Beattie (1989), and plant material was collected as described by Holland et al. (2025). Chemical methods involving ethylenediaminetetraacetic acid (EDTA) soil extractions of Cu and Zn, their ICP-MS measurements, alongside energy-dispersive X-ray fluorescence (ED-XRF) for 15 element concentrations, are included in Supplementary material. EDTA extraction was selected as it is a ligand-based chelating agent of labile, potentially bioavailable pools of Cu and Zn, in line with previous studies at this site (Christie and Beattie, 1989).

### Generation of the microbiological amplicon data

Subsampled fresh soil from each plot was stored at −80 °C for three weeks before DNA extraction. Soil for each of the 48 plots (250 mg) and 1 negative control (no soil) was extracted using Dneasy Powerlyzer Powersoil Kit (Qiagen, Hilden, Germany) according to the manufacturer’s protocol, with the following adaptation: bead beating was done on the Precellys Dual (Bertin Technologies, Montigny-le-Bretonneux, France) for 3 × 15 s at 6,000 rpm. In between cycles, the tubes were placed on ice for 1 min. The extracted DNA was quality-checked on the NanoDrop 8000 spectrophotometer (Thermo Fisher Scientific, Wilmington, Delaware, USA) and via gel electrophoresis alongside a DNA ladder (Thermo Fisher, Vilnius, Lithuania) before it was submitted for amplicon sequencing. Amplicon sequencing was conducted according to the standard Illumina 2-step protocol. Amplicon sequencing of the 16S rRNA gene was conducted via sequencing of the full-length 16S rRNA gene with the PacBio Sequel IIe system. Amplicon sequencing of the ITS was conducted according to the standard Illumina 2-step protocol with target-specific primers for ITS amplicon sequencing [ITS1F/ITS2R (White et al., 1990; Caporaso et al., 2011; Mello et al., 2011)]. On return of the amplicon sequencing data, processing was done according to the QIIME2-2019.04 pipeline (Bolyen et al., 2019). Denoising and generation of amplicon sequence variants (ASVs) were carried out with DADA2 (Callahan et al., 2016). Annotation of the 16S ASV sequences was done against the SILVA database (version 138 SSU) (Quast et al., 2012) and of the ITS ASV sequences against the UNITE database (version 8, 04/02/2020) (Nilsson et al., 2019) using the BLAST consensus option. Following this, the QIIME2-generated count table and taxonomy files were exported for downstream statistical analysis in R version 4.2.2.^1^

### Chemical and microbial statistical analysis

In relation to chemical results, two-way ANOVAs (fixed effects are nutrient treatment, MSS, and subsequent interaction effects) and Dunnett’s ANOVAs (nutrient treatment only) were employed to investigate the soil (Cu, Zn, pH, total carbon) and grass (yield, Cu, Zn) physicochemical attributes. Microbial results were analysed as follows: ASV table and taxonomy file were imported into R studio (R v4.2.2). Phyloseq (version 1.36.0) (McMurdie and Holmes, 2013) was employed to subset the count table at phylum and genus level. Shannon diversity index was calculated from raw ASV-level count data using the estimate richness function and reported in Supplementary Table 3. DESeq2 version 1.32.0 (Love et al., 2014) was used for analysis of the genus-level count table for identification of management differences using the log ratio test (LRT) and WALD test. With respect to the WALD test, results for the following pairwise comparisons were compiled: MSS versus non-MSS were tested, with further calculations done on combined MSS and non-MSS, and on each of the seven nutrient treatments compared separately with the control. ASVs, with overall basemean >50 and adjusted *p*-value <0.001 (LRT test, ~Fertiliser + MSS + Fertiliser * MSS), were deemed as significant. This basemean threshold was applied to eliminate low abundance noise, while the conservative adjusted *p*-value cutoff strictly controlled the false discovery rate across multiple concurrent statistical comparisons.

Following this, only significant ASVs with genus-level annotation were selected. The normalised counts of significant genera plus chemical data of each sample were submitted for Spearman correlation analysis with function rcorr,^2^ generating R and *p*-values. *p*-values were adjusted using the Benjamini–Hochberg false discovery rate (function p.adjust, base R, R core team 2022), and the results from Spearman correlation analysis were integrated into the DESeq2 results table. Subsequently, genera with significant correlations with soil carbon were identified, using the Spearman R-value > 0.5 or < −0.5 as a cutoff for significance. For visualisation of these results, a correlation plot was generated with ggplot2 package and a relative abundance heatmap with function pheatmap.^3^

Canonical Analysis of Principal coordinates (CAP)^4^ was performed to investigate the response of the microbial community to soil treatments with integration of the effect of EDTA Cu, Zn concentrations, pH, and total carbon in the soil across all plots. Permanova (R version 4.4.2; Finkel et al., 2020), a non-parametric test, was employed by function adonis2 (vegan package v2.6–10; Finkel et al., 2020) to test whether MSS management and/or soil nutrient treatments had a statistically significant effect on the soil microbial ITS and 16S genus community, separately. Both analyses were conducted independently at the genus levels using a Bray–Curtis distance metric calculated from all filtered taxa (raw counts greater than 5 in greater than 20% of samples; chloroplast/mitochondria removed) transformed to 1 × 10^6^ proportional abundance. Both the multivariate CAP and permanova analyses were performed on the entire filtered community dataset.

## Results

### Soil chemical characteristics

The following results are considered against our three hypotheses (H_1_–H_3_). PCA analysis (Supplementary Figures 1, 2) reconfirmed previous reports that Cu and Zn were the largest contributors, and organic nutrient group discriminators, to PC 1 and 2 amongst the LTS variables measured. Therefore, relevant to this work are the EDTA extractable Cu and Zn concentrations across the treatments. Both differed significantly by nutrient treatment (*p* < 0.001; Table 1) but not by MSS. As expected, based on the Cu concentrations in the cow slurries, the bioavailability of Cu in the soil was likewise high (Figure 1). The median average in the most intensive slurry application (Cow 200) was ~400 mg/kg. This is an order of magnitude greater than either of the control or NPK treatments, which reported around ~50 mg/kg. The Cu bioavailabilities from the pig slurry-treated plots were lower with no values reported over 150 mg/kg, and far less concentration variation across plots. Also, as expected, Cu increased in a stepwise trend with increasing slurry dosing, irrespective of animal source.

**Table 1:** Variance analysis (ANOVA) results of the soil and grass variables with factors (nutrient treatment, MSS, interaction) of the LTS experiment.

Material	2021 Month	Variable	ANOVA (*F*-value)			Dunnett’s (*T*-value)							
			Nutrient treatment	Multispecies sward	Interaction	Standard Error	NPK	Cow			Pig		
								50	100	200	50	100	200
Soil	Oct	Cu	27.8***	–	–	33.9	–	–	5.2***	12.6***	–	–	3.1*
		Zn	22.9***	–	–	29.9	–	–	–	3.6**	–	5.2***	10.3***
	Feb	pH	27.6***	5.3*	–	0.467	–	–	–	8.9***	–	–	–
		Total C	23.6***	–	–	0.197	–	–	4.2***	10.7***	–	–	3.4**
Grass	Sept	Cu	10.0***	37.2***	3.5**	1.26	–	–	2.9*	2.9*	–	–	4.1**
		Zn	18.3***	41.1***	–	8.59	–	–	–	–	3.1*	2.9*	4.8***
		Yield	29.5***	28.6***	3.7**	483	6.3***	3.5**	5.7***	7.4***	5.3***	7.7***	7.7***

Also, a Dunnett’s comparison of each nutrient application type versus the control treatment. ‘***’ = *p* < 0.001, ‘**’ = *p* < 0.01, ‘*’ = *p* < 0.05. ‘–’ indicates no significant data.

**Figure 1:**
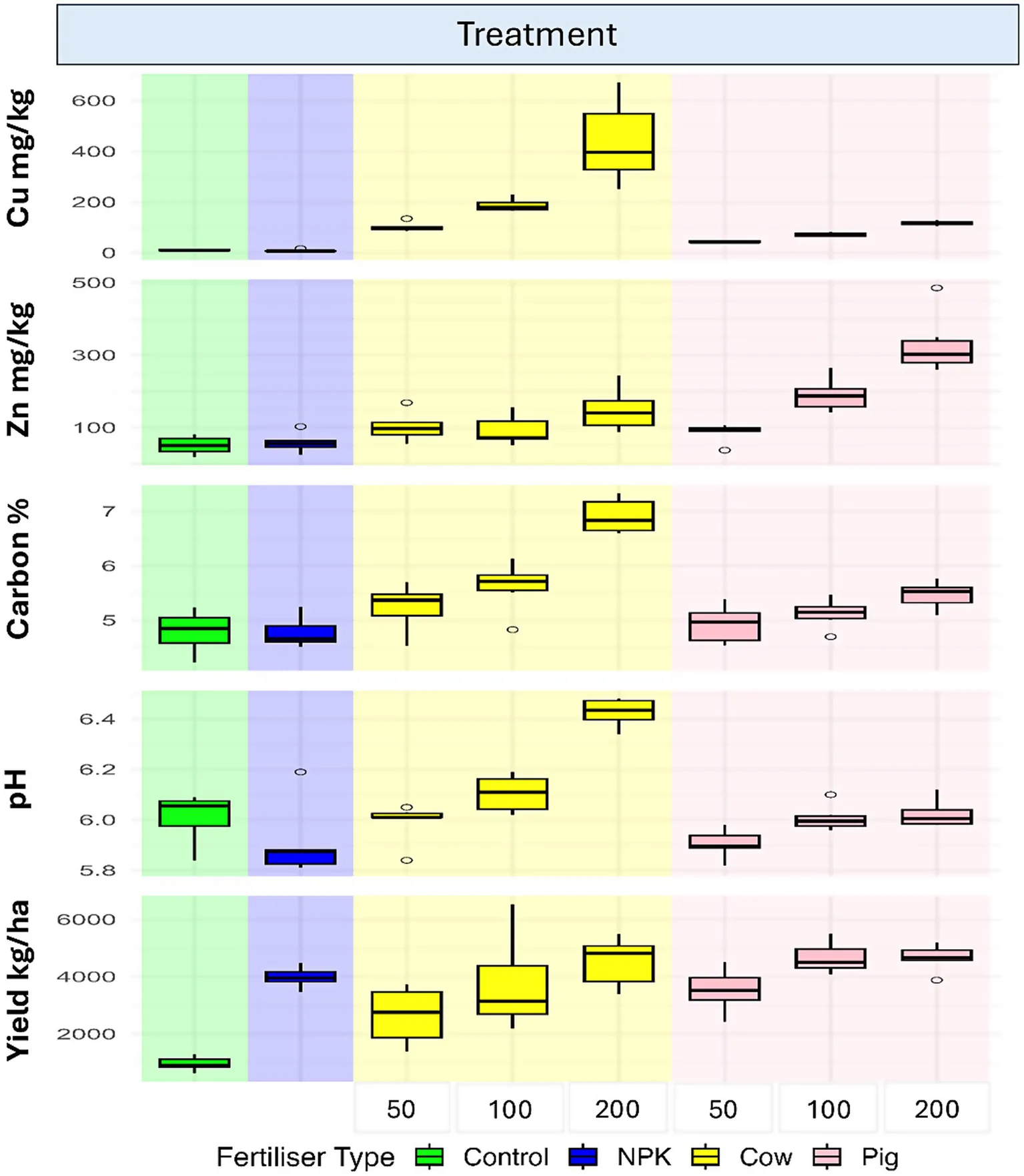
Box plots showing data for treatment soil characteristics. Including concentrations of soil EDTA Cu and Zn, soil total carbon, soil pH, and grass ‘yield’. *N* = 6 per treatment group. Annotations of 50, 100, and 200 denote their m^3^/ha/year application rate. Outliers are denoted by a circle.

Trends for Zn broadly mirror those observed for Cu, with higher treatment loadings again corresponding to greater metal availability. However, the relative contributions of the animal slurries are reversed, with pig slurries exhibiting greater Zn content (Figure 1). The median Zn concentration in soils receiving the highest intensity applications was approximately 300 mg/kg, compared with approximately 50 mg/kg in the control and NPK plots. Other notable chemical patterns were observed for soil total carbon, which differed significantly across nutrient treatments (*p* < 0.001, Table 1). Cow slurries supported the greatest long-term carbon accumulation, with mean total soil carbon reaching ~7%, compared with ~5% in the NPK and control plots (Figure 1). The only major chemical parameter significantly associated with MSS was pH, albeit only at the 0.05 level (*F* = 5.3) (Table 1), with monoculture plots exhibiting lower pH values. As with the other measured parameters, nutrient treatment had a highly significant effect on pH (*p* < 0.001), returning an *F*-value of 28, which falls at the upper end of the range observed across all nutrient-significant treatment parameters (*F* = 23–28) (Table 1).

### Relative bacterial and fungal genus abundances

Long-term nutrient treatment significantly influenced phyla and genus-level community structure in both bacteria (16S) and fungi (ITS) (PERMANOVA; *p* < 0.001; Table 2), with a total of 41 genera meeting the selection criteria thresholds set out in the methods. Nutrient treatment explained a substantial proportion of the variation in community structure (*R*^2^ > 0.66 for both). This provides strong support for H_1_, indicating that long-term nutrient treatment has produced a structurally distinct microbial community. MSS was also a significant driver, albeit with considerably smaller effect sizes (*p* < 0.001 for fungi; *p* < 0.05 for bacteria; *R*^2^ ≈ 0.04 and 0.02, respectively). H_3_ is supported by this, though with a comparatively modest effect size relative to nutrient treatment. There were no archaea nor interactions observed. Permutation test results for CAP (Table 2) showed significant effects for the 16S genus, for all terms; soil Cu, Zn, pH, and combined, but not soil carbon. Cu and Zn were highly significant (*p* < 0.001), pH less so (<0.05) and soil carbon presented at *p* > 0.05. Cu had *F*-values of ~25, followed by Zn (~10), and pH (~3). Fungal changes followed the bacterial significance for Cu and Zn, aside pH and soil carbon which were insignificant. Although the combined explanation of variation trends were both much lower, they also differed between Cu and Zn, with the *F*-values in the latter being marginally higher at ~13 and half as much for ITS Cu at ~12 (Table 2). CAP plots establish marked differences between the bacterial and fungal assemblages (Figure 2). This partially supports H_2_, while both metals significantly structured both community types; Zn showed marginally greater influence on the fungal community relative to Cu, and Cu showed a stronger influence on bacteria.

**Table 2:** Comparison of normalised relative microbial abundance variance in the LTS with experimental factors and soil variables.

Data type	Permanova (R2)		Permutation test for CAP (F)				
	Nutrient treatment	Multispecies sward	EDTA		pH	Soil Carbon	Combined
			Cu	Zn			
Bacteria	0.66***	0.02*	23.5***	10.7***	2.8*	–	12.1***
Fungal	0.66***	0.04***	11.9***	13.3***	–	–	6.9***

Data shows permanova (5,000 permutations) and permutation test for CAP (999 permutations) results for the 16S (bacterial) and ITS (fungal) data for nutrient treatment and MSS, also, soil characteristics (EDTA Cu, Zn and pH) separate and combined. ‘***’ = *p* < 0.001, ‘**’ = *p* < 0.01 and ‘*’ = *p* < 0.05. ‘–’ indicates no significant data.

**Figure 2:**
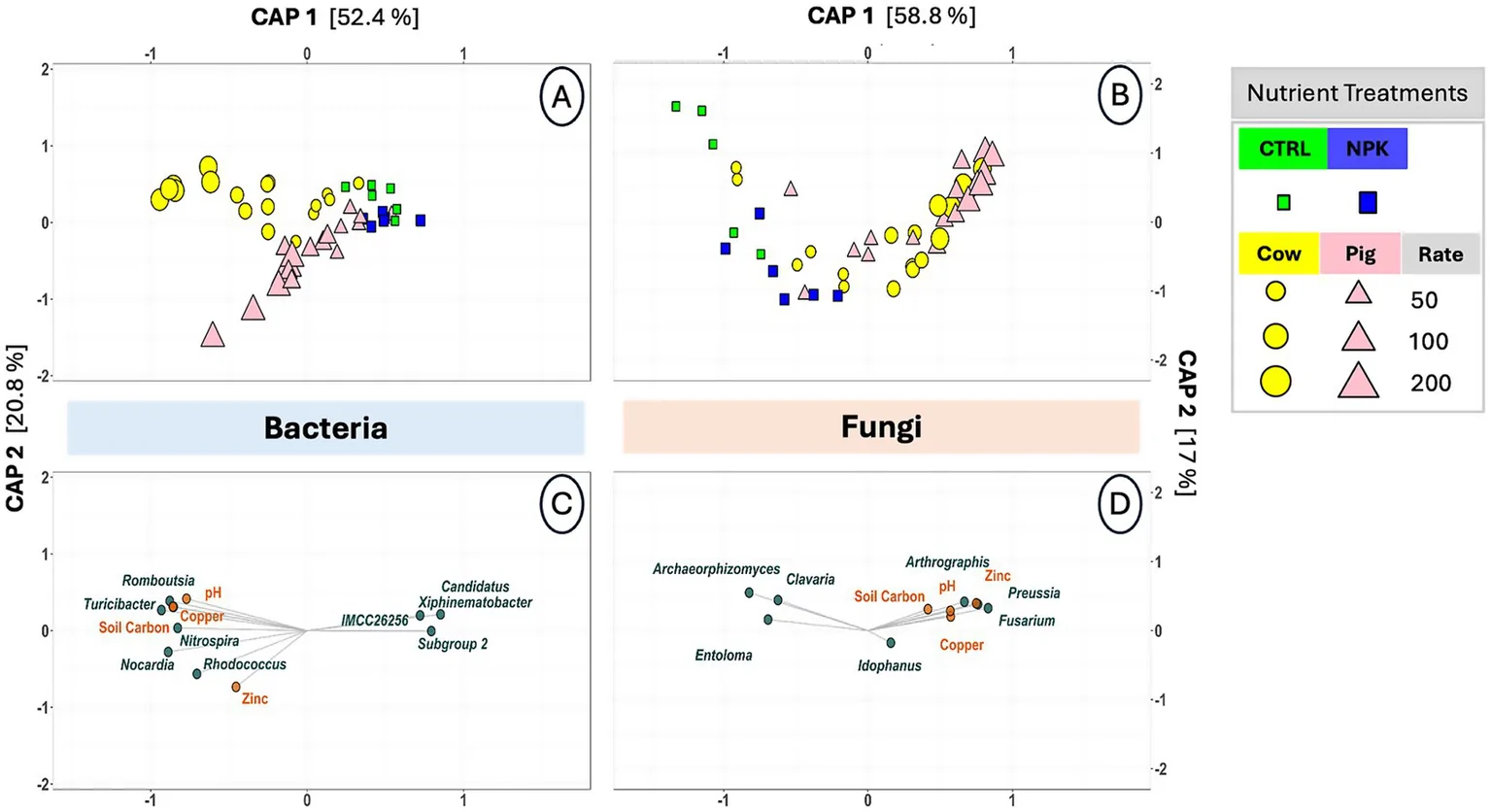
CAP showing separation of relative microbial abundances in the LTS nutrient treatments. Score plots for 16S **(A)** and ITS **(B)** data show separation based on type and rate of nutrient treatment. Score plot shape size is indicative of increased nutrient loading. Loading plots for 16S **(C)** and ITS **(D)** show soil characteristics, EDTA Cu and Zn, pH, carbon and relative microbe abundance. Microbial names are at genus level.

The 16S data differentiated by slurry source and application rate (Figures 2A,C). Cu and pH were the strongest chemical influences for cow slurry-treated plots, while pig slurry-treated plots were discriminated more based on Zn. This distinction between cattle and pig-associated chemical drivers is consistent with H_2_. The control and NPK applications were associated with the lowest application slurries. As rates of manuring increased, bacterial spatial dissimilarities spread. Fungal patterns differed strongly; there were no slurry-source segregations (Figures 2B,D). Here, application rate alone elicited the main response. Also, there were greater differences between the control and NPK treatments and wider spatial dispersion across both CAP1 and 2 axis.

Soil Cu, Zn, pH, and soil carbon combined explained around 52% of the 16S genus microbial community variation on CAP1, and 21% on CAP2 (Figure 2A). Only the low application rate of slurries returned positive CAP1 axis values. The higher application patterns were more complicated. For the cow slurry-amended soils, the medium application rates gave a CAP1 axis range of −0.2 to −0.5. Whereas for the highest application, the values ranged from −0.5 to −1.0. The pig slurry trends at higher application rates were less divergent, with the majority of values falling in the range 0 to −0.25, with the highest being only −0.6.

The microbial clustering patterns, based on relative abundance changes, in the CAP plots were similarly revealing (Figures 2C,D). For bacterial phyla, the low slurry, control, and NPK treatment gave rise to a close grouping of *Actinobacteria*, *Acidobacteriota*, and *Verrucomicrobiota*. As slurry loading increased, the relative abundance patterns altered. A much clearer distribution based on Zn and Cu was observed. This included a cluster of *Firmicutes* that closely associated with Cu and soil carbon. The rest followed a gradient of combined influences from both Cu and Zn factors. The two phyla examples most closely related to Zn were both *Actinobacteria* (Figure 2C). Fungal relative abundance patterns were equally disrupted by the higher slurry additions, with control, NPK, and low applications favouring *Basidiomycota*. As manuring loading increased, relative abundance changes were more pronounced in *Ascomycota* (Figure 2D).

Considering genus, Log-2-fold-change (Log2FC) was used to compare relative abundance changes in the NPK nutrient and highest slurry application treatments with the control, from the WALD test (Table 3). Microbes compared were of the strongest Spearman’s correlations (R) for Cu and Zn. The bacteria that were most negatively affected by slurry application, particularly under cow 200, were *Subgroup 2* (Log2FC −4.27; mean relative abundance declining from 0.76% in the control to 0.04% under cow 200) and *Candidatus Xiphinematobacter* spp. (Log2FC −3.99; 0.47 to 0.03% under cow 200). There was no change noted from the NPK fertilisers. Correlations showed that Cu had the lowest R-value (−0.80) for *Subgroup 2*, and *Candidatus Xiphinematobacter* spp. (−0.81), although for the latter Zn was also moderately strong (−0.58). Conversely, the two bacteria which most increased abundances relatively upon intensive slurry practices were *Nocardia* spp. (Log2FC 9.73; rising from <0.001% in the control to 1.02% under cow 200) and *Nitrospira* spp. (Log2FC 8.25; 0.006 to 1.36% under cow 200). Both showed strong correlations with Cu and Zn, but the R-values were not the highest in the groupings. It was the two *Firmicutes*, *Romboutsia* spp. and *Turicibacter* spp., that were most correlated with Cu with R-values of 0.93 and 0.95, respectively. For fungi, *Archaeorhizomyces* spp. (Log2FC −12.9; mean relative abundance declining from 34.5% in control to 0.004% under pig 200) and *Clavaria* spp. (Log2FC -14.8; declining from 2.35% to <0.001% in pig 200) were most negatively impacted, particularly under pig 200. The positive changes were more complicated. *Fusarium* spp. had high relative abundance increases associated with both cow and pig treatments (Log2FC 8.99 and 9.23, respectively; mean relative abundance rising from <0.001% in the control to 0.26 and 0.12% under cow and pig 200, respectively), while *Preussia* spp. (Log2FC 9.74; rising from 0.004% in control to 0.63% in cow 200) was highest in cow and *Arthrographis* spp. (Log2FC 9.81; <0.001% in the control to 0.08% under pig 200) the highest in pig.

**Table 3:** Microbes with the most notable Spearman’s correlations in the study and their respective abundance changes by nutrient treatment.

Microbe			Changes (Log2FC)			Spearman’s Correlation (R)		
Kingdom	Phyla	Genus	200		NPK	EDTA		Grass Yield
			Cow	Pig		Cu	Zn	
Bacteria	*Acidobacteriota*	*Subgroup 2*	**−4.27**	**−0.92**	–	**−0.80**	−0.49	−0.40
	*Actinobacteria*	*IMCC26256*	−0.41	–	0.35	−0.64	**−0.61**	**−0.46**
		*Nocardia*	**9.73**	**9.55**	–	0.85	**0.69**	0.57
		*Rhodococcus*	5.23	5.98	1.97	0.74	**0.73**	**0.64**
	*Firmicutes*	*Romboutsia*	5.26	2.9	–	**0.93**	0.31	0.36
		*Turicibacter*	7.73	5.93	–	**0.95**	0.46	0.36
	*Nitrospirota*	*Nitrospira*	**8.25**	**7.43**	6.08	0.72	0.60	**0.68**
	*Verrucomicrobiota*	*Candidatus Xiphinematobacter*	**−3.99**	**−3.43**	–	**−0.81**	**−0.58**	**−0.56**
Fungi	*Ascomycota*	*Archaeorhizomyces*	**−10.9**	**−12.9**	–	−0.67	**−0.69**	**−0.67**
		*Arthrographis*	8.5	**9.81**	–	0.66	**0.74**	0.65
		*Fusarium*	**8.99**	**9.23**	5.97	0.66	**0.79**	**0.66**
		*Iodophanus*	6.25	6.5	–	**0.72**	–	**0.66**
		*Preussia*	**9.74**	6.15	−3.4	**0.78**	0.72	0.53
	*Basidiomycota*	*Clavaria*	**−10.94**	**−14.77**	–	**−0.75**	**−0.68**	**−0.62**
		*Entoloma*	−4.91	−3.05	–	**−0.81**	−0.55	−0.51

Microbes selected were the two strongest Spearman’s R correlations with soil EDTA Cu, Zn, and grass yields. Relative abundance changes in Log2FC comparing the control with cow 200, pig 200, and NPK nutrient treatments. ‘200’ denotes 200 m^3^/ha/year. ‘–’ indicates non-significant values. Bold values indicate the strongest two positive and negative R correlations of each microbe, with red and blue the most negative and positive of the metals, respectively.

Across the 41 criteria-selected genera, 25 taxa (bacteria and fungi) increased in relative abundance with slurry applications, whereas 16 declined (Figure 3). The effects of cow-slurry treatments were more prevalent than those of pig slurry. There were clear similar associations between soil carbon and Cu, pH, and, to a lesser extent, Zn in relative correlation strength. Grass yield, unsurprisingly, was highest in the treatments with the greatest nutrient loading and was most positively correlated with *Nitrospira* spp. and *Rhodococcus* spp. among bacteria, and with *Fusarium* spp. and *Arthrographis* spp. among fungi (Table 3). Conversely, *Candidatus Xiphinematobacter* spp. and *IMCC26256* in bacteria, and *Clavaria* spp. and *Archaeorhizomyces* spp. in fungi, exhibited the strongest negative correlations with grass production, i.e., were relatively disadvantaged by high nutrient applications.

**Figure 3:**
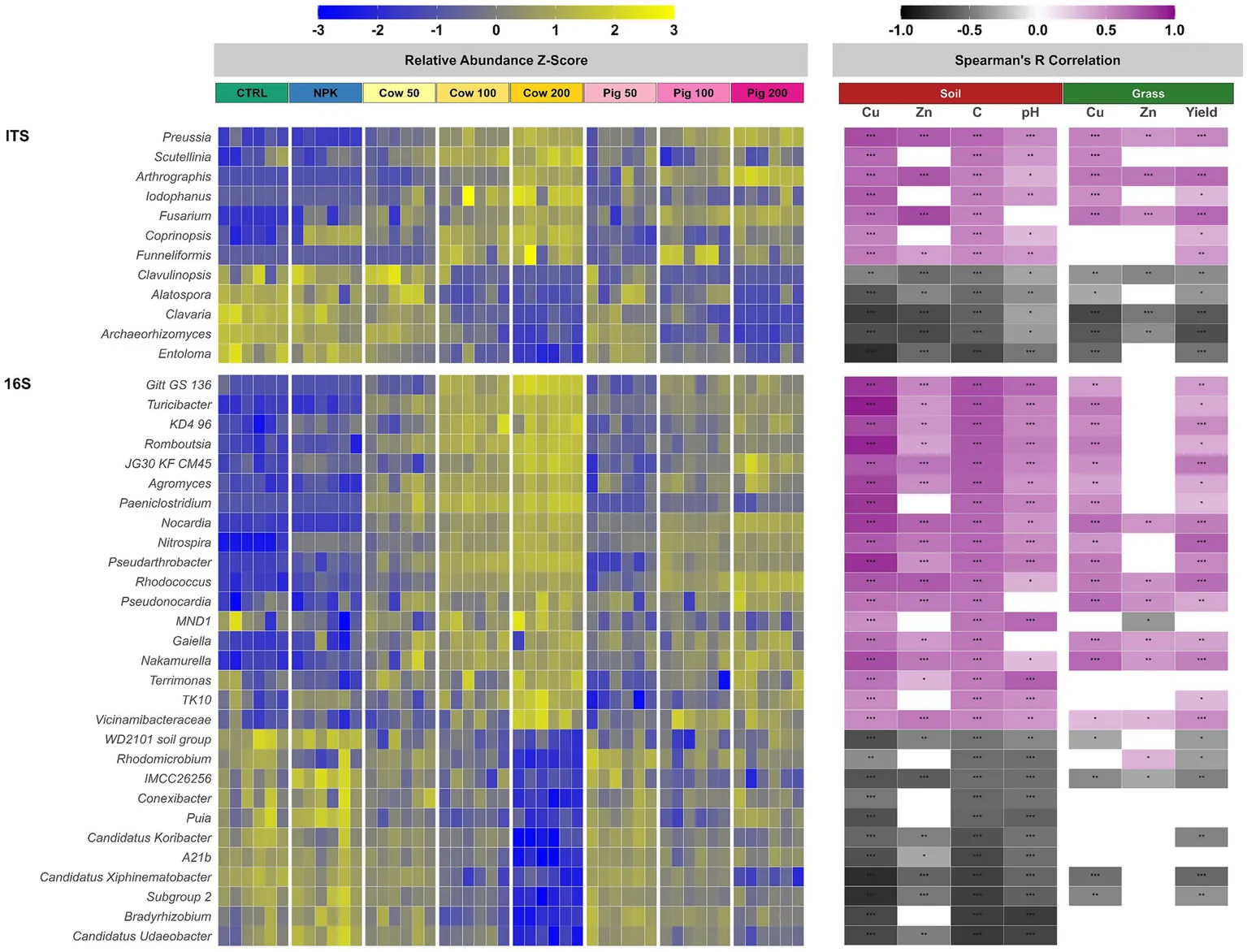
Microbial relative abundances with Spearman’s rank correlations for each LTS treatment and characteristics. Microbial data is arranged in high to low order of R-value correlation to soil total carbon. Microbe names are at genus level. Treatments are annotated as CTRL = no fertiliser treatment, NPK = inorganic fertiliser treatment (200, 32, 160 kg/ha/year), Pig = pig slurry, Cow = cow slurry; slurry application rates for pig and cow slurry are annotated as 50, 100, and 200 to denote m^3^/ha/year. The Z-score scale ranges from deep yellow = 3 indicating higher relative abundances and deep blue = −3 with lower relative abundances, grey = 0 indicates abundances at the microbes overall mean. Spearman’s rank variables are soil EDTA elements (‘Cu’, ‘Zn’), total carbon (‘C’) and ‘pH’, grass ‘yield’ and grass elemental totals (‘Cu’, ‘Zn’). The Spearman’s scale is from dark pink (Spearman’s *R* = 1) to black (spearman’s *R* = −1). White indicates no significant correlation (*p* > 0.05), ‘*’ = *p* < 0.05, ‘**’ = *p* < 0.01 and ‘***’ = *p* < 0.001 of FDR adjusted *p*-values.

## Discussion

Overall, our findings support H_1_–H_3_. Long-term Cu and Zn loadings produced clear structural shifts in microbial community composition, consistent with a settled metal-tolerant community, having established over five decades (H_1_). Community structure also differed by slurry source and application rate, reflecting contrasting Cu and Zn loadings of cattle and pig slurries (H_2_). MSS management was a significant, if comparatively modest, additional driver of community structure (H_3_). The sections below examine genus-level patterns underlying these broader shifts.

There are a number of studies that document the major chemical changes over ~50 years at the LTS experimental plots (Christie, 1987; Christie and Beattie, 1989; Christie and Kilpatric, 1992; Jia et al., 2024). Of these, long-term slurry applications have caused soils to become elevated in Cu and Zn within the organically fertilised plots. These management practices were shown to strongly shape the grassland soil microbial community structure. First reflections of the genera relative abundances seem somewhat counterintuitive, with there being a greater increase in taxa compared to a decline upon Cu and Zn loadings associated with the slurry applications. However, this is understandable given the addition of ruminant and high-carbon-associated bacteria arising with the slurry applications. *Exempli gratia*, the animal-originated, digestive tract-linked bacteria such as *Romboutsia* spp. and *Turicibacter* spp. (Li et al., 2025; Brulin et al., 2025; Fan et al., 2017; Wang et al., 2021) were both detected in the slurry-treated soils. A previous (<10-year) study has found cow- and pig slurry-treated soils to microbially differ from mineral fertiliser, but no difference between the cow and pig (Mora-Salguero et al., 2025). Here, comparison of the cow and pig slurry treatments highlights the greater relative abundances of both the above *Firmicutes* in the cow slurry treatment. This suggests the ruminant microbiome is potentially more influential in modifying the soil microbial community structure, especially over time. This supports H_2_, indicating that manure source shapes community structure through both its distinct microbiome and its differential Cu and Zn loading. Notably, the two *Firmicutes* genera are associated with soil Cu and Zn, which has been found with their phyla in other studies (Li et al., 2022; Sun et al., 2010).

Other bacteria that are known to be metal-tolerant, exhibiting strong correlations with elevated Cu and Zn concentrations, were *Nocardia* spp., a saprophytic soil bacterium, and *Rhodococcus* spp. (Nazari et al., 2022; Shirshikova et al., 2025). The latter reportedly has the ability to withstand metal exposures by biosorbing them to the membrane as a detoxification tool for immobilisation (Nazari et al., 2022). *Nitrospira* spp., a chemolithoautotrophic nitrifier, was also relatively abundant in nutrient-rich treatments of the LTS experiment, which tallies with the higher N additions associated with the maximum slurry amendments (Daims et al., 2015).

A range of fungal genera also responded positively to the nutrient treatments. *Arthrographis* spp., which is abundant where active decomposition occurs, was most abundant in high organic slurry treatments and correlated with increased grass Cu and Zn concentrations and yield (Jongen et al., 2021). *Preussia* spp. was found highly abundant in slurry-amended soils. Its life cycle, which involves spore dispersal via animal ingestion, and its role in organic matter decomposition likely explain its strong association with both metal tolerance and increasing nutrient loading (Gonzalez-Menendez et al., 2017). *Iodophanus* spp. exhibited higher soil Cu and no Zn associations, with the highest relative abundance in medium rates of cow 100 m^3^/ha/year treatments. Notably, this fungus is associated with significantly positive grass yield and thrives in manure- and humus-rich soils (Logo et al., 2026). Of particular interest, *Fusarium* spp. were most relatively abundant in the high slurry application treatments, also showing the strongest positive correlation with zinc of any taxon examined in this study, indicating an enrichment tracking specifically with zinc rather than nutrient treatment. With microbial taxon resolved to genus level, and species identity undetermined, it may be that toxigenic or non-toxigenic species are present. Fusarium infections could lead to mycotoxin contamination, which is a major global health concern (Johns et al., 2022) and potentially be elevated in risk with Zn enrichment from slurry. Silage grass, especially, is vulnerable to mycotoxin contamination and can occur during growth, ensilage, or storage (Zachariasova et al., 2014).

Some bacteria emerged as more prevalent in low-input and low-yield environments. *Subgroup 2* was more abundant in control and NPK treatments, where soil pH was most acidic. While previously suggested to tolerate high metal environments, for example with aluminium, this study did not support such tolerance under Cu and Zn enrichment (Catão et al., 2014). Similarly, *IMCC26256* was associated with low-nutrient, acidic soils and did not respond positively to metals. Instead, its presence reportedly aligns with functions in soil biomass formation and soil structure stabilisation rather than nutrient cycling (Rodriguez et al., 2022). *Candidatus Xiphinematobacter* spp., an indicator of pristine grassland, was previously reported by Armbruster et al. (2021) to increase in relative abundance with soil organic matter and therefore is consistent with its presence in the control treatment. Lastly, some fungi typically associated with nutrient-poor treatments, including *Entoloma* spp., *Clavaria* spp., and *Archaeorhizomyces* spp., were observed primarily in the control treatment. These organisms showed little functional relevance to grass nutrient composition or yield and likely reflect baseline soil biodiversity rather than agriculturally significant activity (Rosling et al., 2011; Venturella et al., 2012; Kumar Sharma and Gautam, 2017).

Although the associations reported here between Cu, Zn, and the microbial community structure are compelling, we interpret them as correlational rather than causal. Cow and pig slurries have altered several soil properties simultaneously, with the relative contribution of each soil variable unable to be fully isolated from these treatments. Slurry contains its own microbiome for each cow and pig input, which has an inoculating effect, with an independence from metal selective pressure over the 50 years. Further, each slurry’s contents can have pharmaceuticals added to them, alongside the copper and zinc which are unaccounted for in this study and may exert their own selection pressure. Due to the nature of this study, and in uncontrollable variables, observed associations may be viewed as consistent with, rather than proof of, metal selection.

## Conclusion

This study highlights the significant impact of long-term slurry applications on soil microbial communities and chemical properties. Three hypotheses concerning the long-term effects of Cu- and Zn-laden slurry application on soil microbial communities were tested, and the findings largely support these predictions. High slurry application rates (200 m^3^/ha/year) enriched some relative microbial genera abundances. These included those involved in nitrogen nutrient cycling, such as *Nitrospira* spp., and potentially pathogenic microbes in the soil, such as *Fusarium* spp. Soil Cu, Zn, and pH were found to significantly alter relative microbial abundances in the soil, leading to changes in bacterial and fungal communities. Multispecies plots that went through soil disturbances caused by tillage and reseeding caused some significant soil pH changes, but did not cause a pronounced impact on the microbial community 2 years after the event. From the obtained results, it is clear that soil copper, zinc, and pH cause significant combined relative changes in soil microbial communities. However, soil pH was not able to alter ITS microbial structure alone.

While high slurry rates provide agronomic benefits through higher yields, they alter soil microbial communities and soil health over the long term. This study revealed that nutrient treatment type does not strongly differ in terms of ITS relative community structure but shows a clear divergence for 16S. Conversely, the amount of nutrient treatment mostly altered the ITS relative community. Careful management of slurry application rates to balance productivity with sustainability is needed as metal accumulation is still occurring in these nutrient treatment plots. These findings underscore the importance of considering soil management practices within a One Health framework, where microbial shifts driven by long-term organic amendments can influence agricultural food systems and cause potential health risks through metal accumulation and mycotoxin-producing fungi. Integrating soil microbiome insights into sustainable nutrient management strategies is therefore critical to maintaining resilient agroecosystems that support One Health.

## Data Availability

The datasets presented in this study can be found in online repositories. The names of the repository/repositories and accession number(s) can be found at: https://www.ebi.ac.uk/ena, ERP158897, PRJEB74207.

## Footnotes

1
www.R-project.org

2 R package corrplot version 0.84, Wei and Simko 2021 DOI: 10.32614/CRAN.package.corrplot.

3 R package pheatmap version 1.0.12, https://cran.r-project.org/web/packages/pheatmap.

4 vegan package v2.6–10, capscale, DOI: 10.32614/CRAN.package.vegan.
