# Supplementary material for: Copper and zinc from slurry applications shape grassland soil microbial community structure in a long-term experiment
Source: Front Microbiol. 2026 Oct 14;17:1930634. doi: 10.3389/fmicb.2026.1930634 (PMC13713379; doi:10.3389/fmicb.2026.1930634)
Supplement: Supplementary file 1 [file Supplementary_file_1.docx]

**Supplementary Information**

**Elemental Quantification**

**Soil ethylenediaminetetraacetic acid extractant**

To maintain continuity with previous LTS experimental studies, we use soil Ethylenediaminetetraacetic acid (EDTA) extractions, prepared as follows. EDTA extractant glass bottle containers were acid washed in 5% v/v analytical grade nitric acid (Scientific Laboratory Supplies, Nottingham, United Kingdom) overnight and washed three times with double distilled deionised water (DDW) (Milli-Q,18.2 MΩ.cm). 14.6 g of trace metal grade EDTA powder (Sigma-Aldrich, St. Louis, Montanna, USA) was mixed with 950 ml DDW (Milli-Q, 18.2 MΩ.cm). Next, 11.2 ml of 25% suprapur ammonia solution (Merck, Darmstadt, Germany) was added to dissolve the EDTA after shaking. A three-point calibrated pH probe (pHenomenal pH1000H, VWR Leuven, Germany) was used to adjust the pH of EDTA liquid to 7 with analytical grade nitric acid (Scientific Laboratory Supplies, Nottingham, United Kingdom). This was then made up to 1L with DDW (Milli-Q, 18.2 MΩ.cm) and mixed.

Freeze dried soil samples of 5g (-/+ 10%) were placed in 50 ml tubes with 25 ml of prepared EDTA extractant, all accurately weighed. Samples were shaken at 1000 speed for 1 hr and left to sit overnight. They were then centrifuged at 600 rfc for 15 minutes. Dilutions made of 167 µl soil-EDTA extractant with 1% nitric acid (v/v) to 10 mL and 5 µl of 10 mg/L rhodium internal standard (Fluka Analytical, Sigma-Aldrich, St. Louis, Montana, USA) were added and precisely weighed. Vortexed tubes were then syringe-filtered with a 0.22 µm Millex MF-Millipore membrane (Merck, Co. Cork, Ireland).

Calibration standards used multi-elemental solution sets at concentrations 0, 0.1, 1, 5, 10, 50, 100 µg/L using a multi-elemental solution of set 2a and 4 (SPEX CertiPrepm Metunchen, New Jersey, USA). A quality control standard was measured intermittently after each 10 samples. Samples were measured in randomised order, and an in-house soil reference material was used in each extractant batch. Measurements were by iCAP TQ-ICP-MS (Thermo Fisher, Bremen, Germany). Limits of detection and relative standard deviations for in-house soil references are in SI Table 1.

**Energy Dispersive X-Ray Fluorescence Measurements**

Dried soil, as used in EDTA extractions, were weighed (3g -/+ 10%) into XRF cups (VWR, Ireland) by a two-point analytical balance (VWR, Italy) and recorded. XRF cups are 32 mm wide, double ended with 4 µm thick prolene (Chemplex, USA) lined at the bottom for X-ray penetration. Samples were hand pressed (Panapress, United Kingdom) with pressure applied for ~20 seconds. Height was recorded by ruler, and sample density was calculated by Rigaku QuantEZ software for elemental concentration quantification. Measurements were performed by Rigaku NEX CG (Japan) benchtop ED-XRF. XRF cups were given a unique identification number and measured in random order with soil certified reference material (CRM) ‘ISE 921’ (Wageningen, The Netherlands) with each XRF run. An XRF run included 7 samples and 1 CRM under a helium atmosphere, with quality control reported in SI Table 1

**Sward Concentrations**

**Inductively Coupled Plasma Optical Emission Spectroscopy**

Plant material is heated in an oven to 80 C for 18hrs, until no change in weight, and milled. Then 250mg (+/-10mg) of plant sample is weighed into digestion tubes, with 5ml nitric acid (69%,AR grade), mixed and left for 2hrs. Two in-house quality control and method blanks are made in each batch of 40. Samples they are securely capped, microwaved (CEM Mars Xpress) as follows: 6 minute ramp to 100 C, hold for 1 minute, ramp to 150 over 10 minutes, hold for 1 minute and 5 minute ramp to 180 C and hold for 20 minutes. After cooling, 45ml of DI water was added and sample added to plastic tubes.

Samples were measured by ICP-OES (Agilent 5100), with a 3 point calibration ranging 0-0.25 mg/L for Cu and 0-0.3 mg/L for Zn. Calibration and QC (0.15mg/L Cu, 0.3mg/L Zn) standards were measured every 5 samples.

**Supplementary Table 1.** Average September 2021 sward data of grass yield (kg/ha), Cu and Zn (mg/kg) alongside standard deviations and number of samples (N). Cow and pig denotations of ‘50’, ‘100’ and ‘200’ indicate m^3^/ha/year of slurry nutrient additions.

| **Sward** | | | | | | | | | |
| --- | --- | --- | --- | --- | --- | --- | --- | --- | --- |
| **Nutrient Treatment** | **Yield** | **Standard deviation** | **N** | **Cu** | **Standard deviation** | **N** | **Zn** | **Standard deviation** | **N** |
| Control | 958.4 | 242.5 | 6 | 7.2 | 0.5 | 6 | 32.1 | 8.0 | 6 |
| NPK | 3980.3 | 347.7 | 6 | 7.3 | 1.0 | 6 | 21.7 | 3.2 | 6 |
| Cow 50 | 2654.2 | 996.0 | 5 | 9.9 | 1.2 | 5 | 36.4 | 8.3 | 5 |
| Cow 100 | 3727.7 | 1626.3 | 6 | 10.9 | 3.9 | 6 | 33.0 | 9.9 | 6 |
| Cow 200 | 4530.4 | 862.4 | 5 | 11.0 | 0.9 | 5 | 32.8 | 9.3 | 5 |
| Pig 50 | 3529.3 | 731.3 | 6 | 8.7 | 1.6 | 6 | 58.8 | 19.0 | 6 |
| Pig 100 | 4659.5 | 540.0 | 6 | 9.3 | 3.0 | 6 | 57.4 | 22.7 | 6 |
| Pig 200 | 4659.6 | 454.4 | 6 | 12.4 | 2.7 | 6 | 73.4 | 22.6 | 6 |

| **Reference Material** | **Element** | **N** | **Certified Value**  **(mg/kg)** | **Recovery**  **(%)** | **Standard Deviation of the Mean** | **Coefficient of Variation (%)** | **L.o.D**  **(mg/kg)** |
| --- | --- | --- | --- | --- | --- | --- | --- |
| **IHRM Soil EDTA**  **(ICP-MS)** | Cu | 5 | - | - | 0.9 | 2.9 | 0.05 |
|  | Zn | 5 | - | - | 3.4 | 3.1 | 7.08 |
| **CRM Soil**  **ISE 921**  **(ED-XRF)** | As | 11 | 29.9 | 116.3 | 1.9 | 5.6 | - |
|  | Ba | 11 | 563 | 98.0 | 18.3 | 3.3 | - |
|  | Ca | 11 | 43000 | 111.1 | 1362.3 | 2.9 | - |
|  | Cu | 11 | 93.8 | 120.3 | 7.2 | 6.4 | - |
|  | Fe | 11 | 31900 | 105.9 | 319.4 | 0.9 | - |
|  | K | 11 | 19100 | 105.3 | 686.6 | 3.4 | - |
|  | Mn | 11 | 1190 | 111.7 | 8.3 | 0.6 | - |
|  | Ni | 11 | 42.4 | 82.2 | 6.7 | 19.1 | - |
|  | P | 11 | 1400 | 110.8 | 51.2 | 3.3 | - |
|  | Pb | 11 | 167 | 117.9 | 3.6 | 1.8 | - |
|  | Rb | 11 | 103 | 115.5 | 1.4 | 1.2 | - |
|  | Si | 11 | 271000 | 94.7 | 2339.1 | 0.9 | - |
|  | Sr | 11 | 165 | 111.3 | 2.5 | 1.4 | - |
|  | Ti | 11 | 3700 | 99.4 | 144.0 | 3.9 | - |
|  | Zn | 11 | 522 | 112.3 | 2.8 | 0.5 | - |
|  |  |  |  |  |  |  |  |

**SI Table 2.** Table showing the In-house Reference Material (IHRM) Quality Control - Soil (County Down, Northern Ireland) (EDTA) and Certified Reference Material (CRM) (ED-XRF). N = number of samples. L.o.D = Limit of Detection.

SI Table 3. Shannon diversity for ITS and16S relative microbial community data. The upper panel shows a two-way ANOVA (Nutrient Treatment, MSS, interaction effects) and lower panel shows mean diversity index with dunnets t-values compared to control. The following symbols are indicated as follows: ‘-‘ = not significant, ‘*’ = P-adj <0.05, ‘**’ = P-adj <0.01, ‘***’ = P-adj <0.001.

| Factor | ITS | | | | 16S | | | |  |
| --- | --- | --- | --- | --- | --- | --- | --- | --- | --- |
|  | F value | P value | DF | Sum of squares | F value | P value | DF | Sum of squares |  |
| Nutrient Treatment | 12.65 | P <0.001 | 7 | 13.24 | 6.88 | P <0.001 | 7 | 0.68 |  |
| MSS | 7.06 | - | 1 | 1.06 | 0.93 | - | 1 | 0.01 |  |
| Interaction | 0.66 | - | 7 | 0.69 | 0.19 | - | 7 | 0.02 |  |
| Nutrient Treatment | Shannon mean | Standard deviation | Standard error | t-value vs Control | Shannon mean | Standard deviation | Standard error | t-value vs Control |  |
|  |  |  |  |  |  |  |  |  |  |
| Control | 3.90 | 0.47 | 0.19 |  | 7.41 | 0.08 | 0.03 |  |  |
| NPK | 4.13 | 0.44 | 0.18 | - | 7.24 | 0.06 | 0.03 | -3.95 ** |  |
| Cow 50 | 4.06 | 0.41 | 0.17 | - | 7.16 | 0.18 | 0.07 | -3.46 ** |  |
| Cow 100 | 4.45 | 0.22 | 0.09 | - | 7.28 | 0.10 | 0.04 | - |  |
| Cow 200 | 4.48 | 0.27 | 0.11 | - | 7.23 | 0.10 | 0.04 | -3.54 ** |  |
| Pig 50 | 3.77 | 0.56 | 0.23 | - | 6.97 | 0.12 | 0.05 | -6.94 *** |  |
| Pig 100 | 2.93 | 0.50 | 0.20 | -4.14 ** | 7.26 | 0.07 | 0.03 | - |  |
| Pig 200 | 3.15 | 0.21 | 0.08 | -3.19 * | 7.29 | 0.14 | 0.06 | - |  |

**SI Figure 1.** Principle component analysis (PCA) score and loading plot of the LTS 0-15cm depth soil. PCA score plot denotes the nutrient treatments groups as ‘Control’ as green, ‘NPK’ as blue, ‘Pig’ ‘50’, ‘100’ and ‘200’ and ‘Cow’ ‘50’, ‘100’ and ‘200’ as progressively deeper shades of pink and yellow, respectively. ‘50’, ‘100’ and ‘200’ denote m^3^/ha/year of slurry nutrient additions. PCA loadings are of ED-XRF soil total concentrations, EDTA Cu and Zn concentrations, Grass Cu, Zn and Yield and Soil C and pH, which are bolded. Orange dots do not feature in the main manuscript, blue dots are of primary importance.

**SI Figure 2.** Total contribution percentage of each loading variable from PC 1 and PC 2 of the PCA presented in **SI Figure 1**.
